# Myelodysplastic syndrome patients present more severe respiratory muscle impairment and reduced forced vital capacity: Is disordered inflammatory signaling the culprit?

**DOI:** 10.1371/journal.pone.0184079

**Published:** 2017-09-06

**Authors:** Bruno Memória Okubo, Anacélia Gomes de Matos, Howard Lopes Ribeiro Junior, Daniela de Paula Borges, Roberta Taiane Germano de Oliveira, Marilena Facundo de Castro, Manoel Ricardo Alves Martins, Romélia Pinheiro Gonçalves, Pedro Felipe Carvalhedo Bruin, Ronald Feitosa Pinheiro, Silvia Maria Meira Magalhães

**Affiliations:** 1 Cancer Cytogenomic Laboratory, Federal University of Ceará, Ceará, Brazil; 2 Post-Graduate Program in Medical Science, Federal University of Ceará, Ceará, Brazil; 3 Department of Clinical and Toxicological Analysis, Federal University of Ceará, Ceará, Brazil; 4 Department of Clinical Medicine, Federal University of Ceará, Ceará, Brazil; Queen's University Belfast, UNITED KINGDOM

## Abstract

**Background/Objectives:**

The ageing process is associated with gradual decline in respiratory system performance. Anemia is highly prevalent among older adults and usually associated with adverse outcomes. Myelodysplastic syndromes (MDS) are a heterogeneous group of hematologic malignancies with increasing incidence with age and characterized by anemia and other cytopenias. The main objectives of this study were to evaluate respiratory muscle strength and lung function in elderly patients with anemia, compare data between myelodysplastic syndromes and non-clonal anemias and evaluate the influence of serum IL-8 level and NF-kB activity on deteriorate pulmonary function in this specific population.

**Participants:**

Individuals aged 60 and older with anemia secondary to MDS, non-clonal anemia and healthy elderly individuals.

**Measurements:**

Forced expiratory volume in 1 second (FEV1), forced vital capacity (FVC), and FEV1/ FVC ratio were measured by spirometry. Respiratory muscle strength was evaluated by maximal static respiratory pressures measurement. IL-8 analysis was performed by ELISA and activity of NF-kB by chemiluminescent assay.

**Results:**

Mean Hb concentration was comparable between patients with anemia. Significant differences were detected between all patients with anemia and controls for maximum-effort inspiratory mouth pressure (PImax) and also for maximum-effort expiratory mouth pressure (PEmax). The MDS group recorded a significantly lower PImax and PEmax percent predicted when compared to non-clonal anemia group. For FVC and FEV1, a significant difference was found in anemic patients, with even significantly lower values for FVC and FEV1 in MDS group. No significant differences were detected for PImax and PEmax and spirometry parameters when anemic patients were stratified according to the degree of anemia. A significant negative impact in FVC (% pred), PImax (% pred) and PEmax (% pred) was observed in patients with MDS and higher levels of IL-8 or increased activity of NF-kB.

**Conclusion:**

A negative impact of anemia, independent of its degree, was demonstrated in respiratory muscle strength and lung function particularly in MDS. The well known elevated proinflammatory cytokines in MDS patients were proposed to play a role as was demonstrated by detrimental effect of higher IL-8 and NF-kB in pulmonary function tests in this population.

## Introduction

Population aging is occurring worldwide with both the number and proportion of older adults increasing globally as fertility rates drop and mortality decreases. In Brazil, the population ageing is growing faster with 13.0% of population currently above the age of 60 years and an expected increase to 30% by 2050 [1].

Typically, the ageing process is associated with gradual decline in respiratory system performance secondary to a varied combination of changes that include lung elastic elements degeneration, loss of parenchymal tissue and gas exchange surface, decrease in chest wall compliance and reduction of intercostal muscle mass and strength [2]. Adaptations taking place within the central nervous system controller help maintain effective gas exchange in these subjects. These complex and interactive effects are reflected by considerable variability in what can be defined as normal respiratory function in the elderly, a matter that is further complicated by the common occurrence of age-related comorbidities [3]. In addition, age-related changes in immune function such as increased circulating pro-inflammatory cytokines, a process formerly described as *inflammaging*, can ultimately be part of the complex mechanisms of gradual loss of muscle mass in aged individuals [4–6]. A variety of molecular pathways could be potentially implicated in this process, including activation of nuclear transcription factor-kappa B (NF-kB) and an increase in its downstream products, such as TNF-alpha and interleukin-8 (IL-8) [7]. NF-kB is part of cells’ auto defense mechanism but its inappropriate activation can mediate inflammation with important role in pathogenesis of several diseases. It triggers the expression of many genes involved in regulating the expression of inflammatory cytokines and pro-survival factors. IL-8 activates multiple intracellular signaling pathways, can cause a wide range of proinflammatory chemical reactions and has been recently proposed as a useful biomarker for various clinical conditions [8]. In chronic obstructive pulmonary disease (COPD), quadriceps muscle strength has been reported to be negatively correlated with plasma IL-8 levels [9] and worse pulmonary function and exercise capacity have been associated with greater plasma concentrations of inflammatory markers, including IL-8 [10].

The World Health Organization (WHO) defined anemia as a hemoglobin (Hb) level below 13 g/dL in men and below 12 g/dL in women, what was further validated through large population studies for elderly people [11]. Anemia is multifactorial and highly prevalent condition among older adults and the frequency increases with age and varies between institutionalized and ambulatory patients. Increasing proinflammatory cytokine expression in this population may play a role. Anemia should not be attributed to senescence, since in most cases an etiological diagnosis can be identified. In common it is usually associated with adverse outcomes, including more frequent hospitalization, disability and poorer survival [12]. Non-clonal anemias due to nutritional deficiencies, chronic inflammation and/or renal insufficiency were identified in two thirds of patients [13]. Between 15 and 30% of anemias in the elderly remained unexplained [13].

Myelodysplastic syndromes (MDS) are a heterogeneous group of hematologic stem cell malignancies characterized by anemia and other cytopenias and an increased risk of transformation to acute myeloid leukemia [14] It is a condition with higher prevalence in individuals aged 65 years or older [15] and the single most common etiology in the last third of older patients with anemia [13]. Disordered modulation of hematopoiesis during aging, accumulation of genomic mutations and abnormal regulation of cytokine production are all contributing factors to the emergence of abnormal clones of hematopoietic cells. Aberrant cytokine expression is believed to contribute to MDS development and/or progression, consistent with the current concept of a direct link between inflammation and oncogenesis.

The main objectives of the study were: (i) evaluate respiratory muscle strength and lung function in elderly patients with anemia and compare data between both myelodysplastic syndromes and non-clonal anemias; (ii) evaluate the influence of serum IL-8 level and NF-kB activity in deteriorating respiratory muscle strength and lung function in this specific population.

## Material and methods

A sample of 35 MDS (Group 1), 33 non-clonal anemia (NC-anemia) (Group 2) and 31 healthy older adults (Group 3) were recruited. This study was approved by Ethics Committee and a written informed consent was provided by all individuals. Regular physical activity, history of chronic pulmonary disease, thoracic surgery, pleural disease, congestive heart failure, neuromuscular disease, kyphoscoliosis, alcohol and drug abuse, current smoking and antecedent smoking with more than 10 pack-years were all exclusion criteria in the study.

MDS was diagnosed by bone marrow puncture and karyotype and was classified according to WHO criteria [16]. Risk stratification was estimated using the Revised International Prognostic Score System (IPSS-R) [17]. The group of non-clonal anemia included older patients with nutritional anemia and anemia of chronic disease.

Resting oxygen saturation was measured using a pulse oximeter (equipment brand Fingertip SB100—Rossmax, China) while the subjects were sitting comfortably. Lung function was assessed by spirometry (Datospir-120c, Sibelmed, Spain) performed according to standard technique [18]. Measurements included forced expiratory volume in 1 second (FEV1), forced vital capacity (FVC), and FEV1/ FVC ratio, and results were compared with previously published normal values for the Brazilian population [19]. Respiratory muscle strength was evaluated by maximal static respiratory pressures measured at the mouth using a manometer (M120, DORMED, Brazil) according to previous recommendations [20].

Maximum-effort inspiratory mouth pressure (PImax) was measured from residual volume and maximum-effort expiratory mouth pressure (PEmax) was measured as close as possible from total lung capacity (TLC). For both measures, at least three trials were performed to obtain a coefficient of variation below 10%. Results were expressed in percentage of predicted values according to American Thoracic Society/European Respiratory Society [20]. All measurements were carried out by the same investigator, using standardized verbal instructions, and were always performed at the same time of the day (between 8:00 and 13:00 p.m.). Each participant completed the Fatigue Severity Scale (FSS) questionnaire to evaluate for the presence and severity of fatigue [21]. Further, the data was divided into subgroups according to severity of anemia (Hb<8.0 g/dL and Hb ≥ 8.0 g/dL).

IL-8 protein concentrations were measured by a commercial ELISA kit (BD OptEIA San Diego, California, USA) according to protocol instructions.

DNA binding activity of NF-kB p65 was measured using TransAM NF-kB p65 kits® (Active Motif, Carlsbad, California, USA), according to the manufacturer’s instructions.

Data analysis used GraphPad Prism for Windows version 6.0 (GraphPad Prism software, La Jolla, CA, USA). Descriptive statistics were calculated for all variables and are reported as means ±1 standard deviation (S.D.). For comparison between two groups the unpaired t test or Mann-Whitney test was used and for comparisons including three groups one-way analysis of variance or Kruskal-Wallis was performed, as appropriate. Post hoc analysis used Bonferroni or Mann-Whitney accordingly. Probability level (*p*-value) <0.05 was adopted as a significance criterion.

## Results

According to IPSS-R, 72% of patients were stratified as lower risk (very low, low and intermediate risk) and 28% as higher risk (high and very high risk). The NC-anemia group was mainly composed of nutritional anemia (iron, cobalamin and folate deficiencies) and anemia of chronic disease. Demographic characteristics of the participants and values for lung function tests are presented in Table 1.

**Table 1:** Physical characteristics and values for pulmonary function tests.

	CONTROLS (mean ± SD)	NC—ANEMIA (mean ± SD)	MDS (mean ± SD)	p
**FVC (%pred.)**	103.8 ± 11.48	76.18 ± 3.37^**a**^	66.09 ± 3.30^**a**^^**b**^	0.000
**FEV1 (%pred.)**	88.32 ± 4.16	68.97 ± 3.09^**a**^	59.00 ± 3.64^**a**^^**b**^	0.000
**FEV1/FVC (L)**	0.77 ± 0.04	0.77 ± 0.05	0.73 ± 0.03^**a**^^**b**^	0.000
**PImax (% pred)**	101.6 ± 8.44	94.03 ± 7.07^**a**^	61.41 ± 10.46^**a**^^**b**^	0.000
**PEmax (% pred)**	103.7 ± 11.34	89.59 ± 4.70^**a**^	64.18 ± 6.96^**a**^^**b**^	0.000
**Hb (g/dL)**	13.46 ± 1.07	8.10 ± 0.75^**a**^	7.56 ± 1.43^**a**^	0.000
**FSS**	24.84 ± 4.20	37.15 ± 9.25^**a**^	37.14 ± 9.00^**a**^	0.000
**SpO2(%)**	94.77 ± 2.23	93.91 ± 2.67	93.60 ± 3.11	0.164

NC—anemia: non-clonal anemia; MDS: myelodysplastic syndrome; % pred: percent predicted; FCV: forced vital capacity; FEV1: forced expiratory volume in one second; PImax: maximal inspiratory pressure; PEmax: maximal expiratory pressure; Hb: hemoglobin concentration; FSS: Fatigue Severity Scale; SpO2: oxygen saturation; BMI: body mass index; SD: standard deviation.

^**a**^ p<0.05 when compared to controls

^**b**^ p< 0.05 when compared to NC-anemia. Kruskal-Wallis test. Post hoc test: Mann-Whitney

There were no significant differences when age was compared between controls and groups 1 and 2 (p = 0.60 and p = 0.19, respectively). Body mass index (BMI) was higher in controls when compared to group 2 (p = 0.01) and comparable between controls and group 1 (p = 0.35). No significant difference between anemic patients was observed (p = 0.41). Mean Hb concentration was comparable between patients with MDS and non-clonal anemia (p = 0.33).

When maximal respiratory pressures were analyzed, significant differences were detected between patients with anemia (groups 1 and 2) and controls for PImax (p<0.001 for both analyses) and also for PEmax (p<0.001 for both analyses). Interestingly, the MDS group recorded a significantly less negative PImax when compared to non-clonal anemia group (p<0.001). A significant difference was also observed for PEmax, with lower values detected in MDS group (p<0.001).

For the spirometric parameters, FVC (% pred) and FEV1 (% pred), a significant difference was found between anemic patients (groups 1 and 2) and controls (p <0.001 for both analyzes of FCV; p <0.001 for both analyzes of FEV1), with lower values in anemic groups. The difference was significant when patients with non-clonal anemia were compared to patients with MDS for FVC (p <0.001) and for FEV1 (p <0.001), with even lower values in MDS group. For FEV1 / FVC (L) there was no difference between controls and NC-anemia patients. Patients with MDS presented significantly lower values when compared to controls and to NC-anemia group. When anemic patients (groups 1 and 2) were stratified according to the degree of anemia (Hb <8.0 g / dL and ≥8.0 g / dL), there were no significant differences for PImax, PEmax or spirometric parameters (Table 2).

**Table 2:** Relationship between lung function parameters and degree of anemia.

Variables	MDS (mean ± SD)			NC—ANEMIA (mean ± SD)		
	Hb <8g/dL	Hb ≥8g/dL	*p-value*	Hb <8 g/dL	Hb ≥8 g/dL	*p-value*
**FVC (% pred.)**	65.90 ± 3.46	66.33 ± 3.11	0.706	75.94 ± 3.62	76.47 ± 3.15	0.665
**FEV1 (% pred.)**	55.90 ± 12.33	60.13 ± 3.66	0.208	69.50 ± 2.99	69.53 ± 3.22	0.348
**FEV1/FVC (L)**	0.76 ± 0.02	0.75 ± 0.02	0.578	0.75 ± 0.03	0.77 ± 0.03	0.054
**PImax (% pred.)**	64.30 ± 9.32	60.53 ± 9.21	0.243	92.83 ± 6.16	94.87 ± 8.24	0.424
**PEmax (% pred.)**	64.85 ± 7.69	63.33 ± 5.71	0.347	89.17 ± 4.70	90.40 ± 4.74	0.460

NC—anemia: non-clonal anemia; MDS: myelodysplastic syndrome; % pred: percent predicted; L: liters; FVC: forced vital capacity; FEV1: forced expiratory volume in one second; PImax: maximal inspiratory pressure; PEmax: maximal expiratory pressure; SD: standard deviation. Mann-Whitney test.

When the presence and severity of fatigue were evaluated using FSS, a significant difference was observed between controls and anemic groups 1 and 2 (p<0.001 for both analyses). The analysis between group 1 and 2 did not show significance (p = 0.99). On average, there was no significant difference in SpO2 among the three groups and saturation was above >90% in all participants (Table 1).

**Fig 1:**
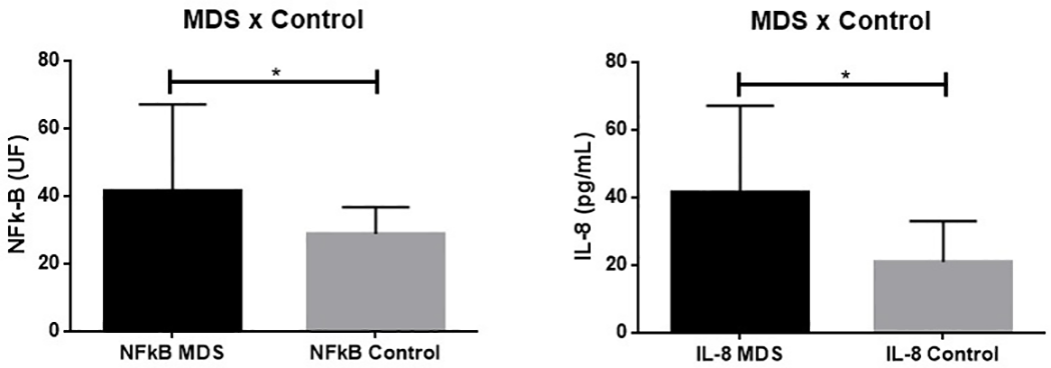
Comparison between NF-kB and IL-8 between MDS patients and controls. *p<0.05.

**Fig 2:**
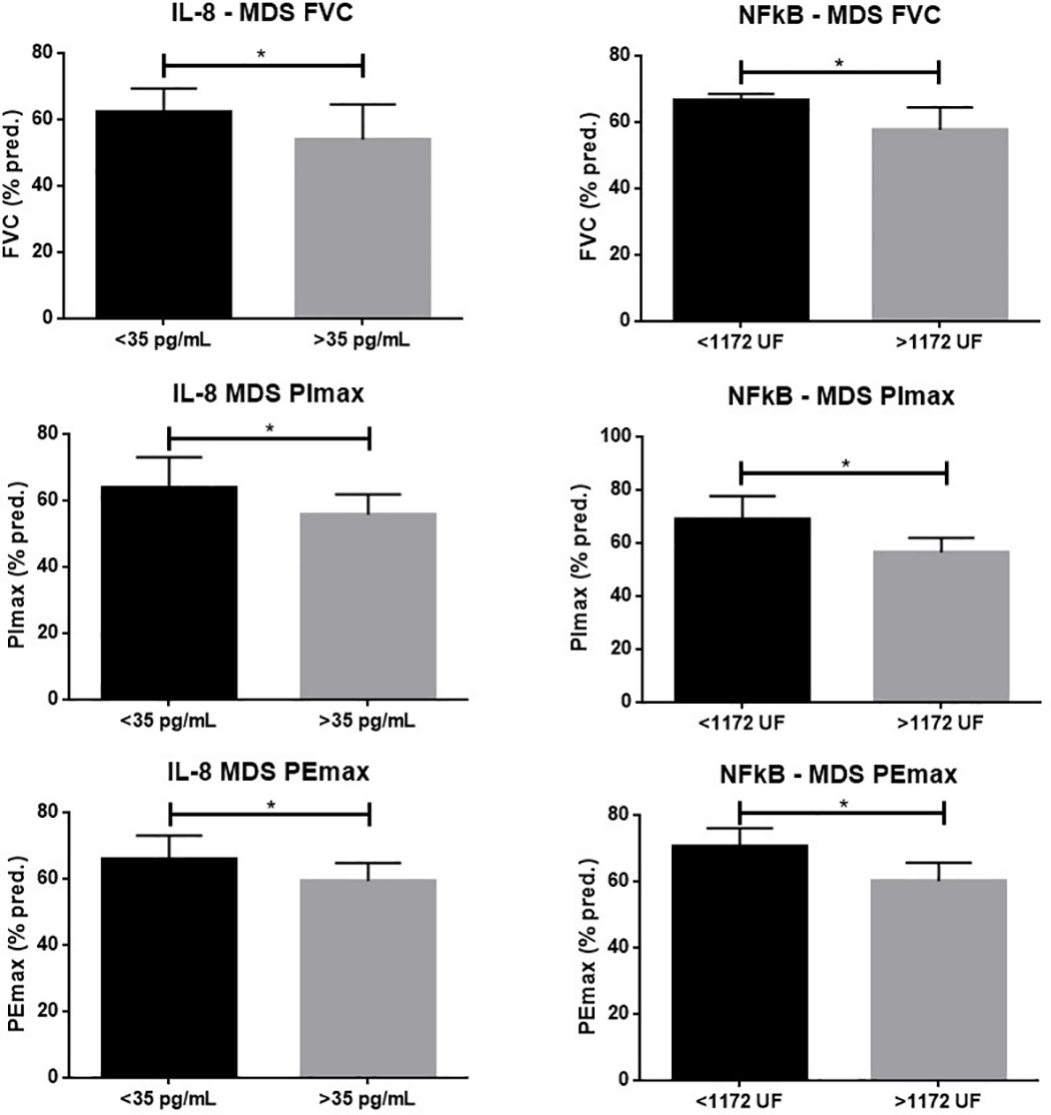
Comparative analysis of forced vital capacity (FVC), inspiratory mouth pressure (PImax), expiratory mouth pressure (PEmax) in patients with MDS according to levels of IL-8 (column A) and activity of NF-kB (column B). Values in percent of predicted. * p< 0.05.

IL-8 levels were significantly higher in MDS patients when compared to controls (p<0.001) as demonstrated in Fig 1. FVC and muscle strength variables according to serum levels of IL-8 are shown in Fig 2. A significant negative impact in FVC (% pred) was observed in patients with MDS and higher levels of IL-8 (p = 0.02). Median was used as a cut-off. Lower values of PImax (% pred) and PEmax (% pred) were also observed in these patients (p = 0.02 for both analysis). No significant difference was observed in healthy subjects when stratified according to the level of IL-8.

NF-kB activity was significantly higher in MDS patients when compared to controls (p<0.001) as demonstrated in Fig 1. The same negative impact was observed when these variables were analyzed according to activity of NF-kB (Fig 2). Higher activity of this factor was significantly associated with lower FVC (% pred), PImax (% pred), PEmax (% pred) (p<0.001 for all analyses). Median was used as a cut-off. No significant difference was observed in healthy subjects when stratified according to the activity of NF-kB.

## Discussion

The effects of ageing on respiratory function are well described and are known to negatively impact physical performance and quality of life [2]. The results of this study highlight the effect of anemia on lung function and respiratory muscle strength in elderly subjects.

On average, maximal respiratory pressures were reduced in patients with anemia compared to controls with normal hemoglobin levels. Moreover, 69% of individuals with non-clonal anemia and 82% of patients with MDS had values for PImax <80% predicted. For PEmax, 57% of individuals with non-clonal anemia and 74% of patient with MDS had values <80% predicted.

These alterations have pathophysiological and clinical relevance since preserved respiratory muscle strength is essential for the generation of the pressure gradient required for ventilation and also for coughing and secretion clearance. As muscle weakness is considered a risk factor for muscle fatigue, a reduction in respiratory muscle strength in these patients also puts them at a greater risk of respiratory muscle fatigue in case of increased respiratory workload [22].

In the present study, anemic patients showed a reduction in FVC and FEV1, when compared to normal controls. These findings are probably secondary to respiratory muscle weakness, as discussed above. Although a reduction in FVC and, consequently, in FEV1, can be present in several conditions, such as lung resection, atelectasis, fibrosis, congestive heart failure, pleural disease and chest wall deformity, these cases were excluded from this study. Obstructive lung diseases can also reduce FVC by limiting deflation of the lungs [23]. However, they are unlikely to have played a role in this study since no individuals with a smoking history of >10 pack-years were included. Although MDS patients showed lower FEV1/FVC ratio, no patient had a FEV1/FVC ratio lower than 0.70. Previously, FVC and FEV1 were found to be reduced in young female subjects with anemia, as compared to controls with normal Hb levels, without any significant difference in FEV1/FVC. In this study, maximal respiratory pressure measurements were not performed [24].

Previous studies have suggested that physical performance can be affected by anemia through various mechanisms, generally related to low oxygen carrying capacity and decreased tissue oxygenation [12, 25]. It has been shown that lower hemoglobin levels can be associated with structural and functional changes in capillarity and muscular fibers [26–27]. A population-based study of 909 individuals aged 65 years and older found that lower hemoglobin levels were associated with lower skeletal muscle strength, lower muscle density, and less muscle mass [28]. As even mild anemia has been associated with worse functional outcomes in the elderly [29], it seems reasonable to expect a similar negative impact on pulmonary performance.

The hematopoietic system of healthy elderly people resembles that of young adults, except for less ability to respond to hematopoietic stress. The pathogenesis of MDS is multifactorial: genetic, epigenetic, apoptotic, and immune factors are involved. Over the last decade, many unraveled specific pathways involved in the complex pathophysiology of MDS have been described. Aberrations in cytokines and their signaling pathways create a permissive milieu for the development of the disease and are a potential key driver of the evolution and progression [30]. Levels of several cytokines and proinflammatory agents have been shown to be elevated in MDS patients compared to healthy controls [31–34].

An important finding of this study was that patients with MSD presented more severe respiratory muscle impairment and forced vital capacity reduction than those with non-clonal anemia group, with comparable degree of anemia. Additionally, we found no significant difference in maximal inspiratory and expiratory mouth pressures and spirometry parameters when anemic patients from both groups were stratified according to the severity of anemia. Expression of TNF-α, IFN-γ, TGF-β, IL-4, IL-6, IL7 and IL-8, among others, has been reported to be deregulated in MDS patients [31]. These inflammatory cytokines are known to induce the production of reactive species of oxygen as well as to deteriorate respiratory muscle function [34]. TNF-α, a proinflammatory cytokine, when administered either *in vitro* or *in vivo* depresses force of respiratory muscles within hours and persists with prolonged exposure. This effect was shown to be opposed by administration of anti-oxidants and preventable by exercise training in mice [35].

NF-kB signaling is considered an important marker since it integrates the intracellular regulation of immune responses in both aging and age-related diseases [36]. Interleukin 8 (IL-8) is currently considered a useful universal biomarker, increasing as a result of many inflammatory conditions [37]. In this study deregulation of immune and inflammatory signaling was demonstrated through significantly higher levels of IL-8 and increased activity of NF-kB in MDS patients with detrimental effect on pulmonary function tests when compared to NC-anemia group.

Considering that mean Hb concentration was comparable between patients with MDS and non-clonal anemia it is reasonable to consider that additional factors, apart from anemia, might be involved. In this population disordered cytokines and inflammatory mediators may have played a role.

Prospective studies are needed to better understand the specific role and mechanisms of inflammation and immunity in MDS, with potential to widen the current scope of therapeutic interventions for this heterogeneous group of diseases.

## Conclusion

Anemia should not be considered as part of the ageing process. Considering its negative impact, it is important to be early diagnosed and treated whenever is possible to prevent anemia-related adverse outcomes. In this study, a negative impact of anemia was demonstrated in respiratory muscle strength and lung function, especially in MDS patients, suggesting that other factors, such as increased cytokines and inflammatory mediators, might be involved. Elevated level of IL-8 and higher activity of NF-kB were associated to deterioration of pulmonary function tests. Taken together, our results provide a rationale for additional studies of cytokines profile and its role in functional respiratory disabilities in higher number of patients.
